# Supplementary material for: AaCaMKs Positively Regulate Development, Infection Structure Differentiation and Pathogenicity in Alternaria alternata, Causal Agent of Pear Black Spot
Source: Int J Mol Sci. 2023 Jan 10;24(2):1381. doi: 10.3390/ijms24021381 (PMC9865007; doi:10.3390/ijms24021381)
Supplement: Supplementary file 1 [file ijms-24-01381-s001.zip › ijms-2091557-supplementary.pdf]

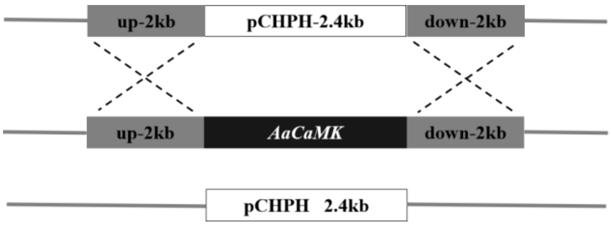

Figure S1. Strategy of homologous recombination knockout of *AaCaMKs*. The target fragment was recovered through a HPH (Hygromycin B) resistance gene cassette and transformed into wild-type protoplasts.

Table S1. Primers used for knockout vector construction.

Gene	Sequence (5′-3′)
AaCaMK1-up	F:ACAGCTATGACCATGATTACGAGCTC GGCGAGGACGGTGAAGAT
	R:GATCCCCGGGTACCGAGCTCGAGCTC TCGGGTCAGCAACAAAGG
AaCaMK1-down	F:TTGCCTAACTCGGCGCGCCGAAGCTTG AGGTGCCATTTCCCAAGT
	R:GTAAAACGACGGCCAGTGCCAAGCTT TATGCGGAGAACGCTGTG
AaCaMK2-up	F:ACAGCTATGACCATGATTACGAATTCCC GCTGACCACGGGTAAGTT
	R:GATCCCCGGGTACCGAGCTCGAATTCC GGAAGTGTTGTAGCAGGAGT
AaCaMK2-down	F:TTGCCTAACTCGGCGCGCCGAAGCTTGA GATGATACAGGAGCAAGAGC
	R:GTAAAACGACGGCCAGTGCCAAGC TTCTAAATGCGAGTGGCAAGAC
AaCaMK3-up	F:ACAGCTATGACCATGATTACGAA TTCGGGGATGTTTGGGTTCAG
	R:GATCCCCGGGTACCGAGCTCGAAT TCTTAGACCGTGCTCTGTTGA
AaCaMK3-down	F:TTGCCTAACTCGGCGCGCCGAAGCT TAGGTATTGTGGCTTGGTTT
	R:GTAAAACGACGGCCAGTGCCAAGCT TGGTCGTGTCGAGGGTAGA

Table S2. The transformants were selected using PDA plates containing 0.08 g L-1 hygromycin B, and then screened by PCR.

Gene	Sequences
AaCaMK1	F: ATGCTCAACAAGCTGCACGG
	R: TCATCGCTTACCCCACAGGC
AaCaMK2	F: ATGGCGACAAGGACTTCGAA
	R: TCATCGCTTACCCCACAGGC
AaCaMK3	F: ATGTCACCCTCCCCTACGCC

	R: CTAACTACCAACCGGCGTCC
OliC	R:ATGTTGGCGACCTCGTATT
TrpC	F: CCCGTAGCAAGGTAGTCAG

Table S3. The primers used for qRT-RCR

Gene	Sequences
AaCaMK1-DL	F: ATACCGCTTCGGAAAGACAC
	R: CTCCAACTCATCGTAGACCA
AaCaMK2-DL	F: CGCCAAGGTCATCAACAAGC
	R: CACCAGCGTCAGATTCGTAA
AaCaMK3-DL	F: CTACACCTCGCAACCTTCTC
	R: GCCAATCTCCTGCTTGACTA
GAPDH	R: CTTACTGCCTCCACCAACTG
	F: TGACGTTGGAAGGAGCGAAG

Table S4. Primers used for complementation vectors.

Gene	Sequences
AaCaMK1-N	F:GCATGGACGAGCTGTACAAGGAGCTCATGCTC AACAAGCT GCACGG
	R:ATGGAGCTATTAAATCACTATCTAGATCACTTC TTCACGGGCTCCG
AaCaMK2-N	F:GCATGGACGAGCTGTACAAGGAGCTCATGGCG ACAAGGACTTCGAA
	R:ATGGAGCTATTAAATCACTATCTAGATCATCGC TTACCCCACAGGC
AaCaMK3-N	F:GCATGGACGAGCTGTACAAGGAGCTCATGTCA CCCTCCCCTACGCC
	R:ATGGAGCTATTAAATCACTATCTAGACTAACTA CCAACCGGCGTCC
N-CX	R:CGACAACCAC TACCTGAGCA
	F:TGAAGGGCGT ACTAGGGTTG
